# Train-your-brain program to reduce depression, anxiety, and stress in stroke survivors: a pilot community-based cognitive intervention study

**DOI:** 10.3389/fneur.2023.1163094

**Published:** 2023-08-10

**Authors:** Xiang Cong Tham, Vanessa Jing Xin Phua, Evelyn Kit Yee Ho, Tingting Yan, Nicole Yun Ching Chen, Lijun Zuo, Claire L. Thompson, Yanhong Dong

**Affiliations:** ^1^Alice Lee Centre for Nursing Studies, Yong Loo Lin School of Medicine, National University of Singapore, Singapore, Singapore; ^2^Nursing Department, Liaocheng Vocational and Technical College, Liaocheng, Shandong, China; ^3^Changi General Hospital, Singapore, Singapore; ^4^Department of Neurology, Beijing Tiantan Hospital, Capital Medical University, Beijing, China; ^5^College of Psychology, Central Queensland University, Rockhampton, QLD, Australia

**Keywords:** depression, anxiety, stress, community intervention, stroke survivors, caregivers, cognitive intervention, brain-training

## Abstract

**Introduction:**

Stroke is a major cause of death and disability worldwide, and it often results in depression, anxiety, stress, and cognitive impairment in survivors. There is a lack of community-based cognitive interventions for stroke survivors. This pilot single trial aimed to assess the feasibility, acceptability, and perceived effectiveness of a community-based cognitive intervention program called Train-Your-Brain (TYB) for stroke survivors and caregivers. The study focused on improvements in emotional and psychological well-being, as well as cognitive functioning.

**Methods:**

A quasi-experimental design was used in this study. A total of 48 participants were recruited and assessed using Depression, Anxiety, Stress Scale – 21 items (DASS-21), Montreal Cognitive Assessment (MoCA) and Symbol Digits Modality Test (SDMT) before and after the intervention. The TYB program consisted of nine sessions and was conducted via the Zoom software application. Participants provided feedback on the program, highlighting areas for improvement.

**Results:**

Twenty-seven stroke survivors and 21 caregivers completed the program. Participants expressed high satisfaction with the TYB program but recommended avoiding assessments in December and customizing the program for stroke survivors and caregivers. Stroke survivors showed significant improvements in depression and stress scores, while caregivers experienced no significant improvements after the program. While there was a slight improvement in stroke survivors’ cognitive scores after the program, it was not statistically significant. Caregivers, however, experienced a significant decline in cognitive scores.

**Discussion:**

The TYB program provided group support and validation, resulting in improved mood and reduced stress among stroke survivors. Cultural collectivism played a significant role in fostering group cohesion. However, the program’s limited focus on caregivers and timing of assessments during the December holidays may have affected the outcomes. The TYB program demonstrated feasibility and potential effectiveness in alleviating psychological distress and enhancing cognitive function among stroke survivors. Future research should explore long-term effects, larger sample sizes, and non-English-speaking populations to enhance generalizability. Tailored interventions for caregivers are necessary.

## Introduction

Stroke may lead to a well-documented range of symptoms such as weakness or paralysis, difficulty with speech or comprehension, vision impairments, and memory loss (1, 2). Hypertension, coronary heart disease, hyperlipidemia, diabetes, and smoking are major risk factors for stroke (3–5). The World Health Organization (WHO) reported that stroke is the second leading cause of death and third leading cause of disability worldwide (6).

In addition to the physical, cognitive, and functional impairments, stroke may also affect an individual’s emotional state and psychological well-being. Mood disorders are common after stroke (7), with a sample of stroke survivors reporting 31% of lifetime depression prevalence and 20% of lifetime anxiety prevalence (8). Irritability, emotional instability, and apathy have also been reported (9). These mood changes may be related to physical limitations, communication difficulties, loss of independence, and changes in social roles (10).

Stroke not only affects individuals who have experienced the event, but it also has a significant impact on their caregivers. Caregiving for a stroke survivor can be physically and emotionally demanding (11) with up to 40% of caregivers of stroke survivors experiencing symptoms of depression and 21% experiencing anxiety (11). Caregivers may face a variety of challenges, such as adjusting to changes in the survivor’s physical and mental abilities, managing caregiving responsibilities while balancing other life demands, and navigating the healthcare system (12). This can result in feelings of overwhelm, isolation, and emotional exhaustion (12). A meta-analysis study found that psychoeducation and social support groups that equip caregivers with problem-solving, caregiving, and stress-coping skills can result in improved psychosocial well-being (13).

While the impact of psychosocial well-being on caregivers of stroke survivors has been explored, the influence of cognitive functioning on these caregivers is less understood. Due to limited literature in this specific area, a literature review on caregivers of individuals with dementia was referenced (14). This review revealed that caregivers, under significant stress, were susceptible to cognitive decline, impairing their ability to provide optimal care. In a similar vein, it is possible that caregivers of stroke survivors may experience suboptimal cognitive functioning due to their caregiving responsibilities. Thus, it is crucial to investigate whether this holds true for caregivers of stroke survivors.

Numerous non-pharmacological approaches have been explored to improve the mood of individuals who have experienced a stroke. These approaches include non-invasive brain stimulation, behavioral therapy or cognitive-behavioral therapy, psychosocial interventions, exercise therapy, acupuncture, light therapy, music therapy, and art therapy (15). A meta-analysis study has indicated that several psychosocial interventions have been found to effectively reduce symptoms of depression, anxiety, and stress in stroke survivors, caregivers, and survivor-carer dyads (16). Moreover, a meta-analysis has indicated that cognitive training has led to cognitive function improvements in healthy older adults (17). Given that stroke survivors are predominantly older adults, and their caregivers are mainly spouses, it would be intriguing to investigate whether caregivers’ cognitive functioning can also benefit (18). While these interventions have demonstrated effectiveness, there is a relative dearth of knowledge compared to the community-based cognitive training program known as Train-Your-Brain (TYB) conducted in Singapore.

Firstly, the TYB program relies on the collectivist attitude prevalent in Singaporean society, which provides participants with social support by forming relationships within the group, enabling them to seek help with each other when needed. Secondly, the TYB program encompasses a dual focus by emphasizing both stroke survivor strategies and active involvement of caregivers in the sessions, facilitating hands-on practice with the stroke survivors. This approach yields threefold advantages: nurturing an enhanced relationship between stroke survivors and their caregivers, providing stroke survivors with practice partners, and potentially benefiting caregivers in terms of their cognitive abilities and mood through the derived program strategies. In contrast, Minshall et al. (16) acknowledged the existence of various definitions of psychosocial interventions in the literature, defining them as interventions comprising psychological and social components. However, the TYB program goes beyond these components by also incorporating cognitive training, which is essential for neurorehabilitation.

Considering the impact of stroke on cognition and psychological well-being for both affected individuals and their caregivers, as well as the growing body of evidence supporting non-pharmacological cognitive interventions post-stroke, the current study aimed to assess the effects of a community-based cognitive intervention for these groups. Specifically, we adapted the Train-Your-Brain (TYB) cognitive health program (19) to examine the feasibility, acceptability, and perceived effectiveness of the intervention. The study aimed to measure emotional and psychological well-being by assessing levels of depression, anxiety, and stress, and improvements in cognitive functioning in both stroke survivors and caregivers.

## Methods

### Study design

This study adopted a quasi-experimental design. The TYB participants were recruited from a community-based brain health for stroke recovery study. These participants were initially recruited from various service providers in Singapore, including day rehabilitation centers, primary healthcare polyclinics, and stroke associations, as well as through word of mouth. There were 64 participants of the brain health study who met the eligibility criteria and were provided with information about this study; 56 of the 64 (87.5%) consented to participate. The criteria for participant eligibility were either: (1) stroke survivors who were at least 3 months post-stroke and who did not have significant aphasia or apraxia that would prevent them from providing informed consent, or (2) any caregivers of stroke survivors. An additional criterion for all participants was sufficient fluency in English to provide informed consent and participate in the group program.

Despite the multicultural nature of Singapore where individuals are proficient in multiple languages, the English language holds a dominant position in the daily lives of its residents. A landscape study conducted on public places in Singapore indicated that English was frequently employed as the primary language of instruction (20). Consequently, considering the study being a pilot, the researchers opted to recruit participants who possessed a good command of the English language.

Before commencement of the program, study team members sent a survey link to the participants and did home visits to obtain baseline assessments. Participants were then invited to attend the program online, conducted via Zoom video conferencing software application. Once the participants completed the program, the study team conducted home visits again for post-intervention assessments. This study obtained institutional ethics approval from the National University of Singapore-Institutional Review Board (NUS-IRB) (NUS-IRB-2021-412).

### Intervention

The TYB program was conducted in English and facilitated by neuropsychologists, with the content based on an earlier version conducted within a public hospital setting (19). The program was delivered over nine sessions with a duration of 60–90 min per session, with two to three sessions scheduled each week. The program design was tailored to align with the cultural context prevalent in Singapore, which predominantly reflects Asian cultural influences.

One of the facilitators, YD, possessed a professional background as a neuropsychologist with 7 years of experience. During YD’s doctoral studies, she conceptualized the Train-Your-Brain (TYB) cognitive health program, which was subsequently employed on older adults in Singapore (19). Another facilitator, NC, was likewise a neuropsychologist with 6 years of professional experience. YD personally guided NC in the implementation of the TYB program to ensure its proper execution. To uphold program fidelity, YD and XT, a health promotion specialist, developed a comprehensive manual outlining the precise execution of each session. YD prioritized attending the sessions facilitated by NC to ensure adherence to the manual’s guidelines. This ensured that the prescribed steps in the manual were faithfully followed during the sessions. Furthermore, YD offered constructive feedback to NC after each session, highlighting areas that could be improved.

Participants who finished the first eight sessions were deemed to have completed the program. Each session focused on a specific topic related to cognition. Upon conclusion of every session, the participants were assigned homework which entailed practical exercises and activities related to the theme of the session, aimed at reinforcing the concepts learnt. The participants were encouraged to discuss as a group on the strategies that they had adopted. Participants were provided with a link to offer feedback on the session they had attended, which they could access at the conclusion of each session. It is important to note that providing feedback was entirely optional.

The TYB session topics were:

#### Session 1: Memory and thinking difficulties related to stroke

The first session of the program focused on providing psychoeducation about the causes and effects of memory and thinking difficulties related to stroke and how to improve it. Participants learnt how to identify these difficulties, understand normal aging and memory processes, and set SMART (Specific, Measurable, Achievable, Realistic/Relevant, and Timed) (21) goals for recovery and brain health. The session also included memory games and a discussion on common memory and thinking problems after stroke among participants. The participants were encouraged to complete the following homework tasks to improve brain health and memory: (1) set a daily goal to improve memory, (2) practice remembering new people’s names, (3) write down three recovery goals for the TYB program, and (4) repeat memory strategies daily.

#### Session 2: Health and lifestyle impact on memory, thinking and stroke recovery

In session two, the facilitator provided an overview of the last session. Participants shared their experiences from the previous session and thereafter, the facilitator provided psychoeducation on the risk factors of recurrent stroke and the importance of healthy lifestyle choices to improve brain health and prevent future recurrence. The goals for this session were to identify common risk factors to brain health and learn techniques for adopting a healthy lifestyle. The session included discussions on the impact of diabetes, heart disease, high blood pressure, high cholesterol, depression and anxiety, obesity, and lack of physical activity on brain health. The homework for participants included setting a SMART goal to improve brain health on a daily/weekly basis. Participants were encouraged to pair up and choose a strategy to implement together. Examples of such goals are getting a health screening, eating more fruits and vegetables, practicing good sleep habits, and avoiding caffeine before bedtime.

#### Session 3: Generating better mood, memory, and recovery

In the third session, the facilitator provided a recap of the previous session and review the group’s homework. The session proceeded with a mindfulness exercise and a discussion of the relationship between depression, anxiety, stress, memory, and thinking difficulties. The facilitator then educated the group on techniques to manage mood and stress to improve memory and set a goal to achieve “Better Mood, Sharper Brain.” The homework assignment asked participants to write a SMART goal to manage their mood and stress for the week and practice their strategies.

#### Session 4: Improving attention, memory, and recovery

The fourth session began with a recap of the previous session and participants sharing their SMART goals and progress to manage mood. The aim of the session was to provide participants with practical skills to enhance their focus and improve their attention. During the session, the facilitator provided psychoeducation about the types of attention, including alertness, divided attention, sustained attention, and selective attention. The facilitator also shared tips on to improve focus: following a routine, choosing the best time for activities, focusing on one task, minimizing distractions, using strategies, being aware of limitations, and managing anxiety. Participants’ homework was to write a SMART goal to improve their focus this week and practice their strategies.

#### Session 5: Learning good planning, and organization skill to optimize recovery

In the fifth session, the facilitator reviewed the important points from the last session, and then focused on the importance of good planning and organizational skills in optimizing recovery. Participants were provided with psychoeducation on how these skills impact thinking and memory, and taught strategies to improve them. The session included a discussion of the different levels of higher-order thinking and the brain structures responsible for them and encouraging participants to set daily goals to achieve a “SMART Plan, Organized Brain.” The homework assignment asked participants to write a SMART goal to help themselves get organized and practice their strategies.

#### Session 6: When to return to work, phased return or full-time work?

After recapping the concepts from the last session, participants received psychoeducation on fatigue related to stroke and learnt ways to cope with it. The session comprised of when to return to work, phased return or full-time work, and the importance of setting SMART goals for return to work and brain health. Participants would have an opportunity to discuss facilitators and barriers for returning to work after stroke. As homework, participants were encouraged to (1) set a daily goal to improve brain health and memory, (2) try to remember three new people’s names, (3) write down three recovery goals to be achieved during the program, and (4) repeat memory strategies, such as SLEEPS [Select a regular bedtime and wake time; Limit use of the bedroom (e.g., no watching TV or using laptop on bed); Exit the bedroom if you are not sleep in 10–15 min; Eliminate naps; Put your feet on the floor at the Same time every morning] (22) for sleep hygiene, daily.

#### Session 7: Manage fatigue, mood, and stress to meet work demand

In session seven, participants started by reviewing the lessons learned from the previous session. Then, the facilitator provided psychoeducation about fatigue, mood, and stress management strategies. Participants were taught about post-stroke fatigue and how to cope with it using cognitive behavioral therapy principles, such as pacing activities, monitoring activity, and engaging in physical exercise to build stamina. The facilitator also discussed how to manage low mood and stress, and participants learnt about physical and behavioral symptoms of depression, as well as techniques to improve mood. Participants were assigned homework to set daily and weekly goals to make small changes to better manage their fatigue, mood, and stress.

#### Session 8: Applying memory and thinking strategies to work effectively

In session eight, the facilitator reviewed the previous session and the participants shared their homework. The session then focused on the application of memory and thinking skills learned so far for work environments. The session included a mindfulness exercise, psychoeducation about mindfulness and its benefits, and a review of the previous lessons on memory and thinking difficulties, health, lifestyle beneficial for brain health, better mood and sharper brain. The facilitator encouraged the group to set goals to apply these skills in the workplace as homework.

#### Session 9: Review session

In the concluding session, the participants were provided with a review of the key takeaways from the previous session, along with an opportunity to discuss their completed homework. Furthermore, the participants were encouraged to share their overall progress and experiences during the program, incorporating the knowledge and concepts that were covered in previous sessions. The facilitator then briefly summarized the salient points covered in each session. Finally, as a final homework assignment, the participants were encouraged to maintain their engagement with the strategies learned throughout the program, even after its completion, to continue to promote growth and improvement.

### Theory of planned behavior

Both stroke survivors and caregivers participated in the same session content as a family unit. While the focus of the program was primarily on meeting the needs of stroke survivors, caregivers were actively encouraged to join the sessions alongside the stroke survivors and engage in practical exercises and activities together. Moreover, caregivers were also encouraged to reinforce the learned concepts by practicing them with their stroke survivors after each session.

The rationale behind the inclusion of caregivers in the TYB sessions stemmed from the utilization of the Theory of Planned Behavior (TPB) proposed by Ajzen (23). This theory primarily centers on individuals who are susceptible to the influence of their social environment, such as friends and family. By employing the TPB, it was anticipated that stroke survivors would be able to observe any modifications in caregivers’ behavior after their participation in the TYB sessions, considering that caregivers are the primary individuals responsible for their care and support. In accordance with the TPB, individuals’ actions or intentions are governed by three fundamental factors: attitude, subjective norms, and perceived control (23).

Attitude refers to an individual’s personal assessment of a health outcome. Attitude consists of two components: experimental and instrumental (23). The experimental component involves the emotional evaluation of a health outcome, whereas the instrumental component encompasses the cognitive evaluation of the expected health outcome and the value attributed to it (23).

The initial session titled “Memory and Thinking Difficulties Related to Stroke” and the sixth session titled “When to Return to Work, Phased Return or Full-time Work?” served as introductory sessions for subsequent topics. The primary objective of the first session was to provide insights into cognitive impairment and its impact on daily functioning. Conversely, the sixth session aimed to shed light on the facilitators and barriers to resuming work. Additionally, these sessions touched upon the positive and negative consequences of both action and inaction in promoting brain health. They also delved deeper into the correlation between pleasure and taking action, particularly in terms of establishing better relationships with their loved ones and caregivers. The ultimate goal of implementing these techniques was to induce behavioral changes among stroke survivors by leveraging the experimental and instrumental components of attitude.

Subjective norms refer to individuals’ beliefs regarding the behavior of their reference groups, encompassing both descriptive norms (beliefs about the behavior exhibited by these groups) and injunctive norms (beliefs about the approval or disapproval of these groups toward specific behaviors) (23). In addition, individuals’ motivation to comply with such behavior stems from their desire to gain the approval of their referent groups.

During sessions 2, 3, 4, 5, 7, and 8, participants were provided with the opportunity to share their experiences and insights regarding the positive outcomes associated with taking action. For instance, in the second session titled “Health and Lifestyle Impact on Memory, Thinking, and Stroke Recovery,” participants were encouraged to discuss the benefits they experienced by incorporating regular exercise into their routines. Furthermore, caregivers were also encouraged to contribute their perspectives. The concluding session in Session 9 solidified the stroke survivors’ beliefs that they were capable of making positive changes based on feedback from group members, and it confirmed the crucial role played by caregivers in supporting their efforts to implement these changes. By employing this technique during these sessions, stroke survivors not only receive encouragement from fellow stroke survivors (descriptive norm), but they also gain knowledge and support from caregivers who are highly supportive of their efforts to make positive changes (injunctive norms).

Perceived control refers to the individual’s belief in their ability to exert control over their behavior. Following each session, participants were assigned homework to practice the strategies taught during the session. The facilitators explicitly instructed participants on how to complete the homework. Caregivers present during the sessions were specifically instructed to encourage stroke survivors to complete the homework and engage in the activities alongside them. Furthermore, at the onset of each session, all participants were encouraged to openly share their personal experiences with others, thereby augmenting the stroke survivors’ perception of control. This collaborative approach aimed to enhance stroke survivors’ confidence in performing positive behaviors.

By employing the TPB, the significance of caregiver participation in the TYB sessions became evident, even though the program was primarily tailored to stroke survivors. Without the involvement of caregivers in the sessions, stroke survivors might lack the intention to make changes in their lives to improve brain health.

### Measurements

Socio-demographic profiles were available from the community-based brain health for stroke recovery study. Before the home visits, participants were asked to complete an online survey which asked about lifestyles, medical adherence, confidence in obtaining employment, quality of life and levels of depression, anxiety, and stress. During the home visits, the participants were evaluated for their cognitive functioning.

#### Depression, anxiety, and stress

The Depression, Anxiety, Stress Scale – 21 items (DASS-21) (24) is a self-reported test that assesses depression, anxiety, and stress by examining 21 items divided into three scales of 7 items each. Scores for each scale are computed by adding the results of the relevant elements. The reliability and validity of DASS-21 are well-established (24–27). Higher scores on the DASS-21 indicate a greater likelihood of experiencing depression, anxiety, and stress.

A research investigation into the cross-cultural applicability of the DASS-21 in Asian countries which include Singapore, revealed that the stress scale is not aligned with the cultural norms and values prevalent in the Asian context (28). For instance, certain items found in the stress scale could be potentially misunderstood as indicating sluggish behaviors within the Asian cultural context. Consequently, the applicability of the DASS-21 within the Asian population may be limited (28). However, it is worth noting that the DASS-21 can still provide a general sense of individuals’ emotional and psychological well-being (29).

#### Cognitive functioning

The Montreal Cognitive Assessment (MoCA) (30) and the Symbol Digits Modality Test (SDMT) (31) were utilized to measure cognitive impairment. While MoCA alone may not be sufficient for determining cognitive impairment, it is recommended to supplement its use with the SDMT (32).

MoCA consists of a 30-point scoring system that incorporates seven distinct cognitive subtests, including visuo-executive functioning, naming, attention, language proficiency, abstraction ability, delayed recall, and orientation acumen. For this study, a modified version of MoCA tailored to the Singaporean population was employed (33). In this modified version, an additional point was added to the total MoCA score for individuals with less than 7 years of education. A higher MoCA score indicates higher cognitive ability.

SDMT is an assessment tool used to evaluate cognitive abilities related to attention, concentration, and processing speed of information. The test involves presenting the participant with a page displaying a top row containing nine symbols, each uniquely associated with digits one through nine. In the subsequent rows, only symbols are shown, and the participant is required to identify the corresponding digit for each symbol. The test duration is limited to 90 s, and a higher score indicates higher levels of cognitive proficiency.

Based on recommendations from a neuropsychologist and insights gained from a literature review (32), the following criteria are employed to evaluate cognitive impairment in an individual: a MoCA score of 23 or lower, and/or an SDMT score of 13 or lower for individuals with 6 years of education or less; and an SDMT score of 32 or lower for those with more than 6 years of education. The utilization of MoCA and SDMT among stroke survivors has received extensive validation (32, 33).

Additional measures were employed to evaluate the practicality of the studies, and these measures relied on self-reports. They included attendance records for each session, participants’ satisfaction with the program rated on a 5-point Likert scale (ranging from “strongly disagree” to “strongly agree”), and written feedbacks provided by the participants. Verbal feedbacks were also obtained during home visits. The tracking of homework assigned to all participants was not implemented, as each participant was given opportunities to share their completed homework at the beginning of each session.

### Statistical analysis

The study employed descriptive analysis to examine the demographic profiles and satisfaction levels of the participants. Furthermore, content analysis was utilized to analyze the open-ended feedback provided by the participants.

Given the small sample size (stroke survivors *n* = 27, and caregivers *n* = 21) in this study, normality was not assumed. Chi-square Test of Independence was conducted to examine the relationships between categorical variables. Mann–Whitney U-test was conducted to compare outcomes between the groups (stroke survivors and caregivers). Wilcoxon Signed Rank Test was conducted to measure the change in cognitive functioning and mood amongst participants within the same group after TYB. The analyses were conducted using Python, version 3.9.12. A two-tailed value of p of less than 0.05 was considered as statistically significant.

A rigorous methodology was employed to ensure comparability and assess the relative performance of cognitive functioning of the participants. The process involved converting the raw scores of individual tests from the Montreal Cognitive Assessment (MoCA) and Symbol Digit Modalities Test (SDMT) into standardized z-scores, leveraging the means and standard deviations (SDs) specific to each test. Subsequently, the scores from the MoCA and SDMT were combined at the individual level. These combined scores were then averaged, resulting in total z-scores. To derive the cognitive composite scores, the total z-scores were further transformed using the composite mean and SD. By implementing this systematic approach, a comprehensive global cognition score was generated for each participant, enabling a comprehensive evaluation of cognitive functioning.

To further examine the intervention’s effect on depression, anxiety, stress, and cognition among participants, the study conducted a *post-hoc* individual analysis. The researchers employed Crawford and Howell’s approach (34) to assess whether notable differences in performance existed between the control sample, used as a reference group, and each individual participant. The control sample participants were matched with each individual based on variables such as age, gender, and education level (35). The established norms, derived from a representative sample treated as the parent population, considered the mean and standard deviation as fixed population parameters rather than estimators. This assessment enabled the researchers to determine whether the performance of the tested individual significantly deviated from what would be expected in a typical population. Additionally, the approach provided an accurate estimation of the proportion of individuals in the general population who might achieve lower scores compared to the observed performance of the tested individual (35). This estimation contributed to a comprehensive understanding of how the individual’s performance compared to others within the overall population (34).

To facilitate individual analyses, a computer software program SINGLIMS.EXE was employed, which can be accessed at the following URL: https://homepages.abdn.ac.uk/j.crawford/pages/dept/SingleCaseMethodsComputerPrograms.HTM#conflims. SINGLIMS.EXE has been specifically developed for comparing an individual’s test score with the score of a normative or control sample (36). This approach utilizes a non-central *t*-test to compare the score of a single case with scores obtained from a control sample. The test determines the extent to which a patient’s score significantly deviates from that of the controls, thereby estimating the abnormality of the patient’s score (i.e., estimating the percentage of the control population with a lower score). Additionally, the test provides associated confidence intervals for this measure. A significant difference of less than 0.05 indicates that the performance of the individual is significantly different from the control sample (35).

## Results

### Socio-demographic profiles

Tables 1, 2 display the socio-demographic profiles of the participants: 56.3% were females; 77.1% were of Chinese ethnicity; the median age was 57.0 ± 22.3; and median years of education was 15.0 ± 3.0.

**Table 1:** Association of socio-demographic profiles and the participants’ categories.

	Stroke survivors (*n* = 27) (OC(EC))	Caregivers (*n* = 21) (OC(EC))	Total (*N* = 48) (*n*(%))	χ^2^ (df)	*p-*value
**Gender**					
Male	16 (11.8)	5 (9.19)	21(43.8)	4.68 (1)	*0.03*
Female	11 (15.2)	16 (11.8)	27(56.3)		
**Ethnicity**					
Chinese	22 (20.8)	15 (16.2)	37(77.1)	0.23 (1)	0.63
Non-Chinese	5 (6.19)	6 (4.81)	11(22.9)		

OC, Observed count; EC, Expected count; χ^2^, Chi-statistics; df, degree of freedom.

**Table 2:** Comparison of socio-demographic profiles between participants’ categories.

	Stroke survivors (median ± IQR)	Caregivers (median ± IQR)	Total (median ± IQR)	*p*-value
Age	59.0 ± 18.5	52.0 ± 27.0	57.0 ± 22.3	0.20
Education	14.0 ± 5.5	15.0 ± 3.0	15.0 ± 3.0	0.98

IQR, Inter-quartile range.

Analysis using Chi-square revealed a significant association between gender and group (stroke survivor; caregiver). More stroke survivors were males, while more caregivers were females [χ^2^ (df = 1) = 4.68, *p* = 0.03]. Other demographic variables did not reveal any significant association with group.

### Feasibility, acceptability, and perceived effectiveness of the TYB program

Eight (14.3%) individuals did not complete the home visit assessments and were therefore excluded from analysis. In total, 48 individuals completed the entire TYB program by successfully attending all eight sessions, thereby meeting the program’s completion criteria. Among these participants, a subgroup of 22.9% (*n* = 11) took part in Session 9, which was designed as a revision session. Out of the 11 participants who attended this session, 6 were stroke survivors.

The written feedback received from participants were mostly positive. There were 162 recorded responses in the feedback forms. Among the 162 respondents, a high percentage expressed agreement and satisfaction regarding various aspects of the sessions. Specifically, 89.5% (*n* = 145) found the sessions interesting, while 88.9% (*n* = 144) indicated that they were easy to understand. A significant majority of 87.0% (*n* = 141) agreed that the sessions successfully achieved all their aims, and 84.0% (*n* = 136) stated that they felt confident in applying the strategies taught during the sessions. Furthermore, 89.5% (*n* = 145) reported enjoying the sessions, and 83.3% (*n* = 135) found them helpful. Most respondents, 88.9% (*n* = 144), agreed that the session contents were sufficient, and a substantial 90.7% (*n* = 147) expressed overall satisfaction with the sessions.

In the study, a total of 20 open-ended feedback responses were collected. Among these, 55.0% (*n* = 11) indicated that the coping strategies, including the utilization of SMART techniques, mindfulness practices, fatigue and stress management, and stress management, were perceived as helpful. Additionally, 10.0% (*n* = 2) of the participants mentioned that every aspect of the sessions proved useful, while the remaining 25.0% (*n* = 7) reported no specific benefits or simply stated “Nil.” In response to inquiries regarding the components of the TYB program that participants did not find valuable, a significant majority of 95.0% (*n* = 19) indicated “Nil,” indicating no specific areas of dissatisfaction. The sole feedback received highlighted the perceived lack of usefulness of the work-related elements within the TYB program. In relation to the inquiry concerning the depiction of memory and thinking strategies that have positively impacted the participants’ lives, findings indicate that a small portion of the participants (10%, *n* = 2) employed the use of mnemonic devices by associating names with specific concepts. Additionally, a larger proportion (35%, *n* = 7) reported the adoption of effective note-taking techniques and the regular practice of setting SMART goals in writing. A minority subset (10%, *n* = 2) emphasized the significance of maintaining sufficient sleep and exercise routines for cognitive enhancement. A single participant (5%, *n* = 1) emphasized the importance of cultivating a positive mindset. A subgroup of participants (15%, *n* = 3) responded with “Nil” when asked about memory and thinking strategies, while the rest responded “Yes.”

A total of 13 verbal responses were documented, from which two themes were derived. Detailed findings derived from the analyzed verbal feedback data are presented in the Supplementary Appendix A.

The second round of home visits took place in December, leading to the formulation of the first theme titled “Challenges of Time Management and Conflicting Priorities, particularly during the month of December,” of which, 23.1% (*n* = 3) expressed as such. This theme emerged due to the observation that December is a period encompassing holidays when individuals often strive to fulfill their year-end objectives. Consequently, participants find themselves involved in numerous activities, as eloquently expressed by one of the caregivers:

*“I've got a ton of stuff on my plate in these upcoming days. I've got to get ready for both Christmas and Chinese New Year, and it's crazy how closely they fall together.”* (P32, 55 years, Female, Caregiver)

84.6% (*n* = 11) of the feedback given by participants expressed the need to customize the TYB program for both stroke survivors and caregivers. This feedback led us to identify the second theme of our research, which we titled “Tailoring TYB and Enhancing Engagement for Stroke Survivors and Caregivers.” It was noted by some participants that the program seemed too generic and did not effectively resonate with certain stroke survivors:

*“The program appears to be appropriate for newly diagnosed stroke patients, although it would be more effective if tailored to different age groups. It feels like a generalized program, but it serves as a necessary foundation. However, some participants might find it a bit tedious, leading to potential loss of interest.”* (P15, 44 years, Male, Stroke Survivor).

Furthermore, caregivers also expressed their opinions regarding tailoring the program specifically for their needs:

*“… I believe it is crucial to include courses specifically designed for caregivers of severely affected stroke survivors, as they require specialized skills and techniques.”* (P32, 55 years, Female, Caregiver)

A possible deviant case which did not fit into the theme, was expressed by a caregiver:

*“I had a really enriching experience during the TYB program with my fellow stroke survivors and caregivers. It truly heightened my awareness and brought us closer together.”* (P31, 70 years, Female, Caregiver)

### Depression

Stroke survivors had significantly higher depression scores (4.0 ± 8.0) than caregivers (0.0 ± 3.0) before the start of TYB (*p* = 0.02) (Table 3). However, there were no significant differences in depression scores between stroke survivors and caregivers after the completion of TYB (Table 4). Table 5 demonstrates a significant improvement in depression scores among stroke survivors, as the scores decreased from 4.0 ± 8.0 to 1.0 ± 6.0 after TYB (*p* < 0.01). Conversely, Table 6 illustrates that there were no significant changes in depression scores among caregivers before and after TYB.

**Table 3:** Comparison of composite cognitive scores, depression, anxiety, and stress scores between participants’ categories before the commencement of Train-Your-Brain program.

	Stroke survivors (median ± IQR)	Caregivers (median ± IQR)	*p*-value
Depression	4.0 ± 8.0	0.0 ± 3.0	*0.02*
Anxiety	4.0 ± 5.5	1.0 ± 3.0	0.09
Stress	4.0 ± 9.5	1.0 ± 3.0	0.07
Cognition	28.2 ± 6.79	37.9 **±** 9.01	*<0.001*

**Table 4:** Comparison of composite cognitive scores, depression, anxiety, and stress scores between participants’ categories after the commencement of Train-Your-Brain program.

	Stroke survivors (median ± IQR)	Caregivers (median ± IQR)	*p*-value
Depression	1.0 ± 6.0	1.0 ± 2.0	0.16
Anxiety	2.0 ± 4.5	1.0 ± 2.0	*0.03*
Stress	2.0 ± 6.5	1.0 ± 4.0	0.38
Cognition	31.5 ± 7.18	28.1 ± 7.23	*0.49*

**Table 5:** Comparison of composite cognitive scores, depression, anxiety, and stress scores in stroke survivors (*n* = 27).

	Before TYB (median ± IQR)	After TYB (median ± IQR)	*p*-value
Depression	4.0 ± 8.0	1.0 ± 6.0	*<0.01*
Anxiety	4.0 ± 5.5	2.0 ± 4.5	0.19
Stress	4.0 ± 9.5	2.0 ± 6.5	*<0.01*
Cognition	28.2 ± 6.79	31.5 ± 7.18	*0.30*

IQR, Inter-quartile range; TYB, Train-Your-Brain.

**Table 6:** Comparison of composite cognitive scores, depression, anxiety, and stress scores in caregivers (*n* = 21).

	Before TYB (median ± IQR)	After TYB (median ± IQR)	*p*-value
Depression	0.0 ± 3.0	1.0 ± 2.0	0.30
Anxiety	1.0 ± 3.0	1.0 ± 2.0	0.13
Stress	1.0 ± 3.0	1.0 ± 4.0	0.49
Cognition	37.9 **±** 9.01	28.1 ± 7.23	<0.001

IQR, Inter-quartile range; TYB, Train-Your-Brain.

Individual analysis indicated that among stroke survivors, 44.4% (*n* = 12) of the general population were expected to attain scores lower than the tested individual’s observed performance. Following the implementation of the program, this proportion decreased to 40.7% (*n* = 11). In contrast, caregivers exhibited a lower proportion of 28.6% (*n* = 6) both before and after the program.

### Anxiety

Stroke survivors generally exhibited higher anxiety scores compared to caregivers, resulting in higher overall scores pre-TYB (Table 3). Although the anxiety scores did not show significant differences between the participant categories, the value of ps were very close to 0.05. Table 4 presents a significant difference in anxiety scores between stroke survivors (2.0 ± 4.5) and caregivers (1.0 ± 2.0) after the completion of TYB (*p* = 0.03). The anxiety scores demonstrated a decrease from pre-TYB (4.0 ± 5.5) to post-TYB (2.0 ± 4.5); however, these changes did not reach statistical significance (*p* = 0.19) (Table 5). Among caregivers, there were no significant changes in anxiety scores before and after TYB, as shown in Table 6.

Among stroke survivors, prior to the program’s implementation, 51.9% (*n* = 14) of the general population were projected to achieve scores lower than the observed performance of the tested individual. Upon introducing the program, this percentage decreased to 37.0% (*n* = 10). Similarly, caregivers initially exhibited a lower proportion of 38.1% (*n* = 8), which decreased to 28.6% (*n* = 6) after participating in the program.

### Stress

Stroke survivors generally exhibited higher stress scores compared to caregivers, although no significant differences were found in stress scores across participant categories, the value of ps were near 0.05, as shown in Table 3. There were no significant disparities detected in stress scores between stroke survivors and caregivers as shown in Table 4. Table 5 demonstrates a significant improvement in stress scores among stroke survivors, with scores decreasing from 4.0 ± 9.5 at pre-TYB to 2.0 ± 6.5 at post-TYB (*p* < 0.01). Conversely, there were no significant changes in stress scores for caregivers before and after participating in the program, as illustrated in Table 6.

In examining stroke survivors, it was found that prior to the program, 44.4% (*n* = 12) of the general population were projected to have scores lower than the tested individual’s actual performance. However, after the program was implemented, this percentage decreased to 37.0% (*n* = 10). In comparison, caregivers initially had a lower proportion of 42.9% (*n* = 9), which further decreased to 33.3% (*n* = 7) following the program.

### Cognitive functioning

In the pre-TYB period, stroke survivors exhibited notably lower composite cognitive scores (28.2 ± 6.79) compared to caregivers (37.9 ± 9.01), with a significant difference (*p* < 0.001) as shown in Table 3. However, there was no significant difference in cognition between the two groups post-TYB. Table 5 reveals an enhancement in cognitive scores for stroke survivors, with an increase from 28.2 ± 6.79 to 31.5 ± 7.18 after TYB; however, the associated value of p is 0.30, indicating no statistical significance. On the other hand, Table 6 demonstrates a significant decline in cognitive scores among caregivers after TYB, as their scores decreased from 37.9 ± 9.01 to 28.1 ± 7.23 (*p* < 0.001).

The individual analysis revealed that, among stroke survivors, 44.4% (*n* = 12) of the general population were expected to have scores lower than the observed performance of the tested individual. After implementing the program, this proportion increased to 51.9% (*n* = 14). Conversely, for caregivers, the initial proportion showing scores lower than the observed performance was 52.4% (*n* = 11), which decreased to 42.3% (*n* = 9) following the program’s intervention. The outcomes derived from individual analyses are available for reference in the Supplementary Appendix B.

## Discussion

The study was a pilot single trial that aimed to assess the feasibility, acceptability, and perceived effectiveness of a community-based cognitive intervention program called Train-Your-Brain (TYB) for stroke survivors and caregivers. The study measured improvements in emotional and psychological well-being, by evaluating levels of depression, anxiety, and stress, as well as cognitive functioning. A total of 48 participants completed the TYB program. The participants in the sessions provided highly positive feedback, expressing satisfaction and agreement with different aspects of the program. However, the participants recommended avoiding the assessments to be conducted in the month of December and customizing the program to better cater to the specific needs of stroke survivors and caregivers. Following the TYB program, the stroke survivors showed significant improvements in depression and stress scores. Caregivers had low levels of depression, anxiety, and stress before and after the program, thus no significant changes were detected. Despite the improvement, the stroke survivors still had higher levels of anxiety than the caregivers after the program. Cognitive functioning scores revealed lower scores in stroke survivors compared to caregivers before the TYB program. While there was a slight improvement in cognitive scores for stroke survivors after the program, it was not statistically significant. Conversely, caregivers experienced a significant decline in cognitive scores after participating in the TYB program. The individual analyses showed that prior to the program, a significant proportion of the general population amongst the stroke survivor participants was expected to have higher depression, anxiety, stress, and lower cognition scores compared to the observed performance of the tested individuals. Following the program’s implementation, there were improvements in these scores among stroke survivors, with a decrease in the proportion of individuals scoring below the expected levels for depression, anxiety, stress scores, and an increase for the cognitive scores. However, among caregivers, there was no change in the proportion of depression scores, whereas there was a noticeable decrease in the proportion of cognition scores following the implementation of the program.

One possible contributing factor to the high levels of depression, anxiety, and stress in the stroke survivors before the TYB program, and the reduced but continued high levels of anxiety after the program, is age-related coping strategies favored by this group. The median age of the stroke survivors was 59.0 ± 18.5, meeting the definition of older persons by WHO (37). A qualitative study conducted with older adults in Singapore found that they tend to utilize avoidant coping mechanisms for managing emotions (38). This can include suppressing their worries by avoiding discussions about them, perceiving non-disclosure to avoid embarrassment, and distracting themselves as a form of escape (38). This may have inhibited the participants’ ability to engage in the group process that is an important component of TYB and to address their anxiety and coping strategies in the TYB forum. Similarly, stroke survivors may be using avoidant coping strategies in daily life, which could contribute to their high levels of depression, anxiety, and stress. In addition, anxiety levels may have remained elevated due to concerns about maintaining positive behavioral changes after the program. Merriman et al. (39) found that stroke survivors often lacked confidence in performing rehabilitation interventions due to limitations caused by the stroke. They may experience difficulties with participating in in rehabilitation and remain anxious about possible long-term outcomes of stroke, which may explain anxiety persisting post-TYB. However, more extensive therapy sessions, including longer relaxation exercises, may be necessary to effectively address anxiety (40). This may have been limited in the current study due to the insufficient number of practical sessions.

The program also provided a platform for participants to receive group support and validation, which are effective in improving mood and reducing stress (40), which explained the significant improvement in depression and stress levels among stroke survivors. This can be largely attributed to the Asian culture. Firstly, there are prevalent stigma and shame surrounding cognitive difficulties in the Asian population (41). The implementation of a group-based cognitive training program was found to be feasible. Secondly, the inclusion of individuals from diverse ethnic backgrounds in the group did not hinder the development of group cohesion or impede mutual support for learning during the program. Lastly, the study indicates that fostering ongoing social support and mutual assistance among group members can contribute to improving cognitive health, with the collectivistic nature of Asian culture playing a significant role in fostering group cohesion.

The significance of cultural collectivism becomes evident as an effective approach to tackle the prevailing isolation resulting from cognitive challenges (41). The vital role of initiating open discussions within the group has emerged as a fundamental strategy in promoting group cohesion and establishing cognitive difficulties as a normal aspect. Consequently, the study proposes explicit recommendations for clinical care, including the facilitator’s early acknowledgment of pertinent Asian cultural factors during group discussions, along with active encouragement of open dialog regarding cognitive difficulties. This approach fosters the formation of a shared cultural identity within the group, ultimately benefiting all participants involved, as evidenced by verbal feedbacks from one of the caregivers (P31).

The scores for depression, anxiety, and stress showed no significant change for the caregivers after the completion of TYB. Nevertheless, individual analyses revealed a slight decrease in the percentage of caregivers with scores higher than the mean scores of the normal population across all measures (except for depression, where no change was observed). Notably, the pre-TYB scores for caregivers were quite low. One possibility, given that most of the caregivers in this study were females, who tend to have a more positive attitude toward seeking professional help for mental health (42–44). Moreover, a considerable number of caregivers were found to be spouses. According to a meta-synthesis study, it was observed that after a year of assuming caregiving roles, these spouses potentially underwent a process of adaptation and recognition toward their newfound responsibilities. (45). This adjustment period potentially led to a significant impact on their personal lives and the amount of time they had available for themselves. Hence, this may explain the low levels of depression, anxiety, and stress in caregivers pre- and post-TYB.

The effectiveness of the TYB program can be attributed to its use of cognitive-based therapy even though only one session focused on psychological and emotional health. While evidence for the benefits of cognitive-based therapy for improving depression, anxiety and stress in stroke survivors is limited, studies on older adults using cognitive-based interventions suggest that improved cognitive functioning leads to improved levels of depression, anxiety, and stress (46–48). It is believed that cognitive impairments in attention/working memory and executive function seen in mood disorders are linked to disruptions in the prefrontal cortex, anterior cingulate cortex, and hippocampus (49). The results suggest that the TYB program’s incorporation of cognitive therapy may have positively impacted the brain structures associated with mood and stress through the process of neuroplasticity.

The cognitive functioning of stroke survivors exhibited a slight improvement, while conversely, caregivers experienced a decline in cognitive functioning subsequent to their involvement in the TYB program. The marginal improvement observed among stroke survivors can be attributed to the timing of data collection, which took place immediately after program completion, thus allowing insufficient time for participants to adequately practice and consolidate the learned skills from the sessions.

In contrast, the decrease in cognitive functioning observed among caregivers in this study aligns with existing literature on the impact of cognitive functioning among caregivers of individuals with dementia (14). The literature review by Fonareva and Oken (14) highlighted the presence of cognitive deficits in these caregivers, including impairments in processing speed, attention, memory, and executive functions. Similarly, it is plausible that some individuals in the caregivers group of this study, as evident in the individual analysis, may have experienced cognitive impairment due to their role in caring for stroke survivors who themselves exhibited cognitive impairment. Fonareva and Oken (14) suggested that caregiver burden and stress could potentially mediate this cognitive decline, although further research is necessary for confirmation. Additionally, the stroke survivors and their caregivers had a symbiotic relationship, wherein the neglect of acute symptoms may have led to chronic adverse consequences (50). Consequently, interventions targeting the stroke survivor-caregiver dyad and addressing the outcomes of both parties may prove beneficial (51). However, in this study, the TYB program primarily focused on stroke survivors, with minimal or no content tailored to caregivers, rendering the strategies less applicable to their specific needs.

Furthermore, caregivers often prioritize meeting the needs of their stroke survivors and making necessary arrangements on their behalf (52), as supported by the participants’ verbal feedback. During the December holidays in Singapore, when cognitive assessments were conducted, caregivers were engaged in activities such as relocation or preparing for festive celebrations. Consequently, future research endeavors will seek to address these limitations and obtain cognitive data that accurately reflects the potential benefits of the TYB program. Specifically, data collection will be scheduled at least 6 months after program completion to allow participants sufficient time to practice the acquired strategies. Moreover, data collection will be planned during non-festive seasons to mitigate potential distractions.

### Limitations

This study had several limitations that must be acknowledged. There was no measurement of the long-term effect of TYB as the post-intervention measures were taken almost immediately on completion of TYB. Hence, the maintenance of gains could not be ascertained. The DASS-21 is not a diagnostic tool for psychological disorders; results may have differed using a clinical diagnostic system and established criteria for diagnosis of depression, anxiety, and stress-related disorders. Since DASS-21 has not been validated on a sample of stroke survivors in Singapore, the applicability of the findings to the broader stroke survivor population might be limited. The small sample in this study did not allow for a control group and randomization, or for blinding of data collectors. The small sample size also reduces the power of the statistical analysis and limits generalizability to a larger population. In addition, the study did not inquire about whether the participants had received any form of social support or counseling from their respective service providers. It is important to consider these factors as these potential mediators could influence the outcomes of the study. Furthermore, this study was limited to only English-speaking participants, thus potentially different outcomes may occur for non-English speaking individuals, which is an important consideration in a multicultural society like Singapore.

### Conclusion

In conclusion, this pilot single trial demonstrated the feasibility and potential effectiveness of the Train-Your-Brain (TYB) program, a community-based cognitive intervention for stroke survivors and caregivers. The program received positive feedback and high levels of satisfaction from participants. To better cater to the specific requirements of stroke survivors and caregivers, it is recommended that the program be customized, and that assessments be avoided in December. The study has also shed light on the importance of age-related coping strategies and cultural collectivism in influencing the outcomes of the program. Future research should consider long-term effects, larger sample sizes, and non-English-speaking populations to enhance generalizability and explore the potential benefits of cognitive-based therapy in stroke rehabilitation. Despite the limitations, these findings indicate that the TYB program holds promise in alleviating psychological distress and enhancing cognitive function among stroke survivors, underscoring the necessity for tailored interventions for caregivers.

## Data availability statement

The original contributions presented in the study are included in the article/Supplementary material, further inquiries can be directed to the corresponding author.

## Ethics statement

The studies involving human participants were reviewed and approved by National University of Singapore-Institutional Review Board. The patients/participants provided their written informed consent to participate in this study.

## Author contributions

XT and YD contributed to conception and design of the study. XT organized the database and performed the statistical analysis. XT wrote the first draft of the manuscript with critical revision from YD. XT, VP, EH, TY, NC, and YD contributed to the data collection and facilitation of the program. All authors contributed to the article and approved the submitted version.

## Funding

This study is funded by Tote Board Enabling Lives Initiatives grant (ref: GC52017NUSBH). YD is a recipient of the Singapore National Medical Research Council (NMRC) Transition Award (TA) award [NMRC/TA/0060/2017].

## Conflict of interest

The authors declare that the research was conducted in the absence of any commercial or financial relationships that could be construed as a potential conflict of interest.

## Publisher’s note

All claims expressed in this article are solely those of the authors and do not necessarily represent those of their affiliated organizations, or those of the publisher, the editors and the reviewers. Any product that may be evaluated in this article, or claim that may be made by its manufacturer, is not guaranteed or endorsed by the publisher.
