# Supplementary material for: Train-your-brain program to reduce depression, anxiety, and stress in stroke survivors: a pilot community-based cognitive intervention study
Source: Front Neurol. 2023 Aug 10;14:1163094. doi: 10.3389/fneur.2023.1163094 (PMC10569939; doi:10.3389/fneur.2023.1163094)
Supplement: Supplementary file 1 [file Data_Sheet_1.docx]

**Appendix A**

| **Themes** | **Participants’ Verbal Feedbacks** |
| --- | --- |
| Challenges of Time Management and Conflicting Priorities especially during December | "I find it irritating to accommodate the programs into my already packed schedule (especially during December 2022).” (P22, 73 years, Male, Stroke Survivor)  “I've been really occupied lately since I recently sold my house back in December 2022. There's a lot of packing to do, which is why I reached out to you (the researchers) to request a change in the timing of the home visit.” (P43, 52 years, Female, Caregiver)  “I've got a ton of stuff on my plate in these upcoming days. I've got to get ready for both Christmas and Chinese New Year, and it's crazy how closely they fall together.” (P32, 55 years, Female, Caregiver) |
| Tailoring TYB and Enhancing Engagement for Stroke Survivors and Caregivers | “In my experience, individual backgrounds and needs vary greatly among stroke survivors and caregivers, which means that the approach to training should also vary. It would be beneficial to offer different levels of courses or modules tailored to the specific needs of different stroke patients. I believe it is crucial to include courses specifically designed for caregivers of severely affected stroke survivors, as they require specialized skills and techniques.” (P32, 55 years, Female, Caregiver)  “The program appears to be appropriate for newly diagnosed stroke patients, although it would be more effective if tailored to different age groups. It feels like a generalized program, but it serves as a necessary foundation. However, some participants might find it a bit tedious, leading to potential loss of interest. The aspect of identifying what to focus on and what to avoid, such as alcohol, was particularly helpful and could be summarized.” (P15, 44 years, Male, Stroke Survivor).  “The sessions allowed us to explore more advanced techniques specifically tailored for stroke survivors. It's important to note that not all of us experienced memory issues; some of us faced physical weaknesses instead. The training we received varied in intensity, aiming to enhance our memory skills. One helpful strategy was teaching seniors to utilize their mobile devices for memory-related tasks, such as relying on the calendar app to manage all our appointments effectively.” (P10, 59 years, Female, Stroke Survivor)  “The TYB session is quite valuable, especially when it comes to engaging in discussions. However, individuals suffering from speech aphasia may encounter challenges in actively participating and contributing to the conversation. Nevertheless, I believe it serves as a beneficial resource, particularly for stroke patients who find themselves alone, as it provides them with an opportunity to acquire coping strategies and learn how to navigate their circumstances.” (P30, 56 years, Female, Caregiver)  “Session is beneficial, the duration is satisfactory, yet the pace induces anxiety, especially for stroke patients who become fidgety and tense. Introverted stroke participants may prefer a conducive environment that encourages open discussion, allowing them to speak up when they feel comfortable. It would be valuable to provide caregivers with a shareable PowerPoint presentation (edited version, acknowledging that not all content can be shared). Stroke participants are unsure how to utilize the handout provided, so interactive elements like fill-in-the-blanks could enhance engagement.” (P33, 40 years, Female, Caregiver)  “Engaging in body sense and mindfulness exercises is beneficial for stroke patients. However, it would be even more advantageous if the video speed were reduced to 20%.” (P38, 68 years, Male, Caregiver)  “In my opinion, the Zoom sessions felt too short. I suggested having longer durations, especially when more participants join. It would be great if we could split the session into a training segment followed by a discussion segment. This way, those who are willing to stay can continue the session while those who need to leave can do so without any issues. What I found particularly fascinating about these Zoom sessions were the valuable insights shared by individuals based on their personal experiences.” (P26, 66 years, Male, Stroke Survivor)  "The sessions were quite lengthy, but I must admit that I have certainly acquired valuable skills and techniques." (P41, 46 years, Male, Caregiver) |
| - | “I had a really enriching experience during the TYB program with my fellow stroke survivors and caregivers. It truly heightened my awareness and brought us closer together.” (P31, 70 years, Female, Caregiver) |

“-” means no theme assigned

**Appendix B**

Stroke Survivors (N=27)

|  | **Depression** | **Anxiety** | **Stress** | **Cognition** |
| --- | --- | --- | --- | --- |
| *Baseline* |  |  |  |  |
| Mean | 5.33 | 4.00 | 6.11 | 29.0 |
| Standard Deviation | 5.40 | 4.42 | 6.12 | 5.01 |
| *Follow-up* |  |  |  |  |
| Mean | 3.63 | 3.30 | 3.85 | 29.7 |
| Standard Deviation | 4.68 | 3.44 | 4.39 | 6.29 |

| **Participant ID** | **Depression Score** | **% of population below case’s score**  **(Lower Limit,**  **Upper Limit)** | **Anxiety Score** | **% of population below case’s score**  **(Lower Limit,**  **Upper Limit)** | **Stress Score** | **% of population below case’s score**  **(Lower Limit,**  **Upper Limit)** | **Cognition Score** | **% of population below case’s score**  **(Lower Limit,**  **Upper Limit)** |
| --- | --- | --- | --- | --- | --- | --- | --- | --- |
| *Baseline* | | | | | | | | |
| P1 | 7 | 61.8%  (46.8%,  75.6%) | 4 | 50.0%  (35.3%,  64.7%) | 0 | 16.8%  (7.28%,  29.9%) | 27.7 | 40.5%  (26.5%,  55.5%) |
| P2 | 2 | 27.5%  (15.3%,  42.1%) | 3 | 41.3 %  (27.2%,  56.2%) | 4 | 36.9%  (23.3%,  51.9%) | 27.4 | 37.8%  (24.1%,  52.8%) |
| P3 | 6 | 54.8%  (39.9%,  69.2%) | 3 | 41.3 %  (27.2%,  56.2%) | 4 | 36.9%  (23.3%,  51.9%) | 36.0 | 91.0%  (80.6%,  97.3%) |
| P4 | 9 | 74.5%  (60.0%,  86.3%) | 6 | 67.0%  (52.1%,  80.1%) | 11 | 78.0%  (64.0%,  89.0%) | 36.2 | 91.0%  (80.6%,  97.3%) |
| P5 | 7 | 61.8%  (46.8%,  75.6%) | 7 | 74.5%  (60.0%,  86.3%) | 7 | 55.6%  (40.7%,  70.0%) | 32.8 | 77.0%  (62.8%,  88.2%) |
| P6 | 16 | 96.8%  (90.6%,  99.6%) | 10 | 90.3%  (79.5%,  97.0%) | 13 | 86.0%  (73.7%,  94.6%) | 35.6 | 89.6%  (78.5%,  96.6%) |
| P7 | 12 | 88.2%  (76.6%,  95.8%) | 11 | 93.4%  (84.3%,  98.4%) | 14 | 89.1%  (77.9%,  96.4%) | 26.6 | 31.9%  (19.0%,  46.8%) |
| P8 | 0 | 17.1%  (7.44%,  30.2%) | 0 | 19.1%  (8.88%,  32.7%) | 0 | 16.8%  (7.28%,  29.9%) | 21.8 | 8.46%  (2.41%,  18.6%) |
| P9 | 0 | 17.1%  (7.44%,  30.2%) | 0 | 19.1%  (8.88%,  32.7%) | 0 | 16.8%  (7.28%,  29.9%) | 33.8 | 82.4%  (69.2%,  92.2%) |
| P10 | 0 | 17.1%  (7.44%,  30.2%) | 5 | 58.7%  (43.7%,  72.8%) | 4 | 36.9%  (23.3%,  51.9%) | 36.5 | 92.3%  (82.6%,  98.0%) |
| P11 | 0 | 17.1%  (7.44%,  30.2%) | 0 | 19.1%  (8.88%,  32.7%) | 0 | 16.8%  (7.28%,  29.9%) | 25.2 | 23.1%  (11.8%,  37.2%) |
| P12 | 0 | 17.1%  (7.44%,  30.2%) | 0 | 19.1%  (8.88%,  32.7%) | 7 | 55.6%  (40.7%,  70.0%) | 32.5 | 75.3%  (61.0%,  87.0%) |
| P13 | 2 | 27.5%  (15.3%,  42.1%) | 0 | 19.1%  (8.88%,  32.7%) | 0 | 16.8%  (7.28%,  29.9%) | 24.8 | 21.0%  (10.2%,  34.8%) |
| P14 | 18 | 98.5%*  (94.5%,  99.0 %) | 2 | 33.0%  (19.9%,  47.9%) | 14 | 89.1%  (77.9%,  96.4%) | 19.9 | 4.38%  (0.78%,  11.8%) |
| P15 | 14 | 93.6%  (84.7%,  98.5%) | 10 | 90.3%  (79.5%,  97.0%) | 12 | 82.3%  (69.1%,  92.1%) | 27.8 | 41.3%  (27.2%,  56.2%) |
| P16 | 0 | 17.1%  (7.44%,  30.2%) | 0 | 19.1%  (8.88%,  32.7%) | 0 | 16.8%  (7.28%,  29.9%) | 28.2 | 44.0%  (29.7%,  58.9%) |
| P17 | 2 | 27.5%  (15.3%,  42.1%) | 0 | 19.1%  (8.88%,  32.7%) | 4 | 36.9%  (23.3%,  51.9%) | 23.4 | 14.3%  (5.65%,  26.7%) |
| P18 | 4 | 40.5%  (26.5%,  55.5%) | 5 | 58.7%  (43.7%,  72.8%) | 2 | 25.8%  (13.9%,  40.2%) | 23.0 | 12.5%  (4.53%,  24.3%) |
| P19 | 7 | 61.8%  (46.8%,  75.6%) | 4 | 50.0%  (35.3%,  64.7%) | 7 | 55.6%  (40.7%,  70.0%) | 28.4 | 45.4%  (31.0%,  60.3%) |
| P20 | 8 | 68.4%  (53.6%,  81.3%) | 4 | 50.0%  (35.3%,  64.7%) | 8 | 61.8%  (46.8%,  75.6%) | 27.8 | 41.2%  (27.1%,  56.2%) |
| P21 | 3 | 33.7%  (20.5%,  48.7%) | 0 | 19.1%  (8.88%,  32.7%) | 5 | 43.0%  (28.8 %,  57.9%) | 20.6 | 5.56%  (1.18%,  14.0%) |
| P22 | 8 | 68.4%  (53.6%,  81.3%) | 18 | 99.8%*  (98.7%,  100%) | 20 | 98.3%*  (93.8%,  99.9%) | 31.5 | 68.8%  (54.0%,  81.6%) |
| P23 | 13 | 91.2%  (80.9%,  97.4%) | 7 | 74.5%  (60.0%,  86.3%) | 20 | 98.3%*  (93.8%,  99.9%) | 31.0 | 64.9%  (50.0%,  78.3%) |
| P24 | 3 | 33.7%  (20.5%,  48.7%) | 4 | 50.0%  (35.3%,  64.7%) | 2 | 25.8%  (13.9%,  40.2%) | 29.6 | 54.7%  (39.8%,  69.1%) |
| P25 | 4 | 40.5%  (26.5%,  55.5%) | 5 | 58.7%  (43.7%,  72.8%) | 7 | 55.6%  (40.7%,  70.0%) | 30.9 | 64.3%  (49.3%,  77.8%) |
| P26 | 0 | 17.1%  (7.44%,  30.2%) | 0 | 19.1%  (8.88%,  32.7%) | 0 | 16.8%  (7.28%,  29.9%) | 36.9 | 93.5%  (84.4%,  98.4%) |
| P27 | 0 | 17.1%  (7.44%,  30.2%) | 0 | 19.1%  (8.88%,  32.7%) | 0 | 16.8%  (7.28%,  29.9%) | 26.6 | 32.5%  (19.5%,  47.4%) |
| *Follow-up* | | | | | | | | |
| P1 | 1 | 29.3%  (16.7%,  44.0%) | 0 | 17.8%  (7.93%,  31.1%) | 0 | 19.8%  (9.40%,  33.5%) | 31.7 | 62.6%  (47.6%,  76.2%) |
| P2 | 0 | 22.6%  (11.5%,  36.7%) | 1 | 25.9%  (14.0%  40.4%) | 1 | 26.5%  (14.4%,  41.0%) | 28.0 | 39.8%  (25.9%,  54.8%) |
| P3 | 2 | 36.7%  (23.1%,  51.7%) | 1 | 25.9%  (14.0%  40.4%) | 0 | 19.8%  (9.40%,  33.5%) | 33.0 | 69.5%  (54.8%,  82.3%) |
| P4 | 7 | 75.7%  (61.4%,  87.3% | 7 | 85.0%  (72.4%,  93.9%) | 4 | 51.3%  (36.5%,  65.9%) | 39.2 | 92.5%  (82.9%,  98.0%) |
| P5 | 6 | 68.9%  (54.0%,  81.7%) | 7 | 85.0%  (72.4%,  93.9%) | 7 | 75.6%  (61.3%,  87.2%) | 36.8 | 86.4%  (74.1%,  94.8%) |
| P6 | 17 | 99.5% **  (97.7%,  100%) | 11 | 98.2%*  (93.6%,  99.8%) | 10 | 91.0%  (80.5%,  97.3%) | 31.5 | 61.2%  (46.2%,  75.1%) |
| P7 | 12 | 95.5%  (87.9%,  99.2%) | 11 | 98.2%*  (93.6%,  99.8%) | 14 | 98.4%*  (94.2%,  99.9%) | 38.9 | 91.9%  (81.9%,  97.8%) |
| P8 | 2 | 36.7%  (23.1%,  51.7%) | 0 | 17.8%  (7.93%,  31.1%) | 1 | 26.5%  (14.4%,  41.0%) | 19.7 | 6.56%  (1.57%,  15.7%) |
| P9 | 0 | 22.6%  (11.5%,  36.7%) | 0 | 17.8%  (7.93%,  31.1%) | 0 | 19.8%  (9.40%,  33.5%) | 36.0 | 83.5%  (70.5%,  92.9%) |
| P10 | 0 | 22.6%  (11.5%,  36.7%) | 5 | 68.5%  (53.6%,  81.4%) | 2 | 34.1%  (20.8%,  49.0%) | 34.2 | 75.5%  (61.2%,  87.1%) |
| P11 | 0 | 22.6%  (11.5%,  36.7%) | 2 | 35.7%  (22.2%,  50.7%) | 0 | 19.8%  (9.40%,  33.5%) | 19.5 | 6.23%  (1.44%,  15.1%) |
| P12 | 0 | 22.6%  (11.5%,  36.7%) | 2 | 35.7%  (22.2%,  50.7%) | 6 | 68.3%  (53.4%,  81.2%) | 33.5 | 72.3%  (57.7%,  84.5%) |
| P13 | 0 | 22.6%  (11.5%,  36.7%) | 3 | 46.7%  (32.2%,  61.5%) | 0 | 19.8%  (9.40%,  33.5%) | 16.2 | 2.24%  (0.23%,  7.38%) |
| P14 | 14 | 98.1%*  (93.4%,  99.8%) | 1 | 25.9%  (14.0%  40.4%) | 5 | 60.0%  (45.0%,  74.0%) | 19.0 | 5.44%  (1.14%,  13.8%) |
| P15 | 7 | 75.7%  (61.4%,  87.3% | 5 | 68.5%  (53.6%,  81.4%) | 8 | 81.9%  (68.5%,  91.8%) | 31.8 | 63.0%  (48.0%,  76.6%) |
| P16 | 0 | 22.6%  (11.5%,  36.7%) | 0 | 17.8%  (7.93%,  31.1%) | 0 | 19.8%  (9.40%,  33.5%) | 28.5 | 42.6%  (28.4%,  57.6%) |
| P17 | 1 | 29.3%  (16.7%,  44.0%) | 3 | 46.7%  (32.2%,  61.5%) | 3 | 42.5%  (28.3%,  57.5%) | 25.3 | 25.1%  (13.4%,  39.5%) |
| P18 | 0 | 22.6%  (11.5%,  36.7%) | 1 | 25.9%  (14.0%  40.4%) | 0 | 19.8%  (9.40%,  33.5%) | 23.9 | 19.0%  (8.81%,  32.6%) |
| P19 | 6 | 68.9%  (54.0%,  81.7%) | 4 | 57.9%  (42.9%,  72.0%) | 6 | 68.3%  (53.4%,  81.2%) | 33.5 | 72.3%  (57.7%,  84.5%) |
| P20 | 6 | 68.9%  (54.0%,  81.7%) | 2 | 35.7%  (22.2%  50.7%) | 3 | 42.5%  (28.3%,  57.5%) | 28.2 | 40.9%  (26.8%,  55.9%) |
| P21 | 1 | 29.3%  (16.7%,  44.0%) | 0 | 17.8%  (7.93%,  31.1%) | 1 | 26.5%  (14.4%,  41.0%) | 22.6 | 14.0%  (5.46%,  26.3%) |
| P22 | 4 | 53.1%  (38.2%,  67.6%) | 10 | 96.7%  (90.2%,  99.5%) | 15 | 99.0%*  (96.0%,  100%) | 35.4 | 80.9%  (67.4%,  91.2%) |
| P23 | 5 | 61.2%  (46.2%,  75.0%) | 4 | 57.9%  (42.9%,  72.0%) | 8 | 81.9%  (68.5%,  91.8%) | 29.2 | 46.9%  (32.4%,  61.8%) |
| P24 | 1 | 29.3%  (16.7%,  44.0%) | 3 | 46.7%  (32.2%,  61.5%) | 1 | 26.5%  (14.4%,  41.0%) | 28.6 | 43.7%  (29.5%,  58.7%) |
| P25 | 6 | 68.9%  (54.0%,  81.7%) | 6 | 77.6%  (63.6%,  88.7%) | 8 | 81.9%  (68.5%,  91.8%) | 32.1 | 64.7%  (49.7%,  78.1%) |
| P26 | 0 | 22.6%  (11.5%,  36.7%) | 0 | 17.8%  (7.93%,  31.1%) | 1 | 26.5%  (14.4%,  41.0%) | 36.0 | 83.5%  (70.5%,  92.9%) |
| P27 | 0 | 22.6%  (11.5%,  36.7%) | 0 | 17.8%  (7.93%,  31.1%) | 0 | 19.8%  (9.40%,  33.5%) | 28.6 | 43.7%  (29.5%,  58.7%) |

* <0.05, ** < 0.01

Caregivers (N=21)

|  | **Depression** | **Anxiety** | **Stress** | **Cognition** |
| --- | --- | --- | --- | --- |
| *Baseline* |  |  |  |  |
| Mean | 2.05 | 1.76 | 2.95 | 37.8 |
| Standard Deviation | 2.94 | 2.32 | 4.07 | 5.32 |
| *Follow-up* |  |  |  |  |
| Mean | 1.57 | 1.14 | 2.38 | 29.7 |
| Standard Deviation | 2.29 | 1.31 | 2.62 | 6.25 |

| **Participant ID** | **Depression Score** | **% of population below case’s score**  **(Lower Limit,**  **Upper Limit)** | **Anxiety Score** | **% of population below case’s score**  **(Lower Limit,**  **Upper Limit)** | **Stress Score** | **% of population below case’s score**  **(Lower Limit,**  **Upper Limit)** | **Cognition Score** | **% of population below case’s score**  **(Lower Limit,**  **Upper Limit)** |
| --- | --- | --- | --- | --- | --- | --- | --- | --- |
| *Baseline* | | | | | | | | |
| P28 | 7 | 94.2%*  (84.2%,  99.1%) | 4 | 82.1%  (66.8%,  93.0%) | 5 | 68.6%  (51.7%,  83.0%) | 37.1 | 44.9%  (28.7%,  61.7%) |
| P29 | 2 | 49.4%  (32.9%,  66.0%) | 3 | 69.6%  (52.8%,  83.8%) | 1 | 32.2%  (17.7%,  49.1%) | 30.4 | 9.42%  (2.33%,  21.8%) |
| P30 | 2 | 49.4%  (32.9%,  66.0%) | 1 | 37.6%  (22.3%,  54.6%) | 1 | 32.2%  (17.7%,  49.1%) | 44.4 | 87.8%  (74.2%,  96.3%) |
| P31 | 0 | 25.2%  (12.1%,  41.6%) | 1 | 37.6%  (22.3%,  54.6%) | 0 | 24.3%  (11.5%,  40.7%) | 31.8 | 14.0%  (4.65%,  28.3%) |
| P32 | 0 | 25.2%  (12.1%,  41.6%) | 0 | 23.4%  (10.8%,  39.6%) | 2 | 41.1%  (25.3%,  58.0%) | 38.3 | 53.5%  (36.7%,  69.8%) |
| P33 | 10 | 99.2%*  (96.1%,  100%) | 9 | 99.7%*  98.0%  100% | 15 | 99.6%*  (97.4%,  100%) | 39.4 | 61.3%  (44.3%,  76.8%) |
| P34 | 0 | 25.2%  (12.1%,  41.6%) | 1 | 37.6%  (22.3%,  54.6%) | 0 | 24.3%  (11.5%,  40.7%) | 37.8 | 49.5%  (33.0%,  66.1%) |
| P35 | 0 | 25.2%  (12.1%,  41.6%) | 0 | 23.4%  (10.8%,  39.6%) | 0 | 24.3%  (11.5%,  40.7%) | 24.9 | 1.39%*  (0.05%,  5.94%) |
| P36 | 0 | 25.2%  (12.1%,  41.6%) | 0 | 23.4%  (10.8%,  39.6%) | 0 | 24.3%  (11.5%,  40.7%) | 34.0 | 24.5%  (11.6%,  40.8%) |
| P37 | 0 | 25.2%  (12.1%,  41.6%) | 0 | 23.4%  (10.8%,  39.6%) | 1 | 32.2%  (17.7%,  49.1%) | 37.2 | 45.5%  (29.4%,  62.3%) |
| P38 | 2 | 49.4%  (32.9%,  66.0%) | 3 | 69.6%  (52.8%,  83.8%) | 3 | 50.5%  (33.9%,  67.0%) | 33.4 | 21.5%  (9.43%,  37.4%) |
| P39 | 0 | 25.2%  (12.1%,  41.6%) | 0 | 23.4%  (10.8%,  39.6%) | 3 | 50.5%  (33.9%,  67.0%) | 41.5 | 74.7%  (58.3%,  87.8%) |
| P40 | 7 | 94.2%*  (84.2%,  99.1%) | 5 | 90.6%  (78.2%,  97.7%) | 8 | 88.0%  (74.5%,  96.5%) | 36.7 | 41.6%  (25.7%,  58.5%) |
| P41 | 0 | 25.2%  (12.1%,  41.6%) | 0 | 23.4%  (10.8%,  39.6%) | 0 | 24.3%  (11.5%,  40.7%) | 44.0 | 86.5%  (72.4%,  95.6%) |
| P42 | 0 | 25.2%  (12.1%,  41.6%) | 0 | 23.4%  (10.8%,  39.6%) | 0 | 24.3%  (11.5%,  40.7%) | 37.9 | 50.2%  (33.6%,  66.7%) |
| P43 | 1 | 36.6%  (21.4%,  53.6%) | 0 | 23.4%  (10.8%,  39.6%) | 6 | 76.4%  (60.1%,  89.0%) | 43.8 | 85.8%  (71.5%,  95.2%) |
| P44 | 0 | 25.2%  (12.1%,  41.6%) | 0 | 23.4%  (10.8%,  39.6%) | 0 | 24.3%  (11.5%,  40.7%) | 32.1 | 15.1%  (5.30%,  29.7%) |
| P45 | 3 | 62.3%  45.3%  77.6% | 1 | 37.6%  (22.3%,  54.6%) | 0 | 24.3%  (11.5%,  40.7%) | 43.0 | 82.3%  (67.0%,  93.1%) |
| P46 | 5 | 83.1%  68.0%  93.6% | 4 | 82.1%  (66.8%,  93.0%) | 11 | 96.6%  (90.0%,  99.7%) | 44.4 | 87.8%  (74.2%,  96.3%) |
| P47 | 0 | 25.2%  (12.1%,  41.6%) | 3 | 69.6%  (52.8%,  83.8%) | 3 | 50.5%  (33.9%,  67.0%) | 43.7 | 85.4%  (71.0%,  95.0%) |
| P48 | 4 | 73.8%  (57.3%,  87.1%) | 2 | 53.9%  (37.2%,  70.2%) | 3 | 50.5%  (33.9%,  67.0%) | 38.7 | 56.1%  (39.2%,  72.2%) |
| *Follow-up* | | | | | | | | |
| P28 | 8 | 99.4%*  (96.7%,  100%) | 1 | 45.8%  (29.6%,  62.6%) | 5 | 83.0%  (67.9%,  93.6%) | 32.0 | 64.0%  (47.1%,  79.2%) |
| P29 | 0 | 25.5%  (12.4%,  42.0%) | 0 | 20.3%  (8.60%,  36.1%) | 0 | 19.2%  (7.88%,  34.8%) | 21.1 | 9.83%  (2.51%,  22.4%) |
| P30 | 5 | 92.0%  (80.5%,  98.3%) | 1 | 45.8%  (29.6%,  62.6%) | 2 | 44.4%  (28.3%,  61.3%) | 34.9 | 78.9%  (63.0%,  90.8%) |
| P31 | 0 | 25.5%  (12.4%,  42.0%) | 1 | 45.8%  (29.6%,  62.6%) | 1 | 30.6%  (16.4%,  47.4%) | 19.4 | 6.18%  (1.07%,  16.5%) |
| P32 | 0 | 25.5%  (12.4%,  42.0%) | 0 | 20.3%  (8.60%,  36.1%) | 0 | 19.2%  (7.88%,  34.8%) | 28.1 | 40.6%  (24.9%,  57.6%) |
| P33 | 3 | 72.5%  55.9%  86.1% | 3 | 90.9%  (78.6%,  97.8%) | 6 | 90.4%  (78.0%,  97.6%) | 31.5 | 61.2%  (44.2%,  76.7%) |
| P34 | 0 | 25.5%  (12.4%,  42.0%) | 1 | 45.8%  (29.6%,  62.6%) | 2 | 44.4%  (28.3%,  61.3%) | 27.6 | 37.7%  (22.4%,  54.7%) |
| P35 | 0 | 25.5%  (12.4%,  42.0%) | 0 | 20.3%  (8.60%,  36.1%) | 0 | 19.2%  (7.88%,  34.8%) | 21.8 | 11.7%  (3.41%,  25.1%) |
| P36 | 0 | 25.5%  (12.4%,  42.0%) | 0 | 20.3%  (8.60%,  36.1%) | 0 | 19.2%  (7.88%,  34.8%) | 27.9 | 39.0%  (23.5%,  56.0%) |
| P37 | 1 | 40.5%  (24.8%,  57.5%) | 1 | 45.8%  (29.6%,  62.6%) | 1 | 30.6%  (16.4%,  47.4%) | 27.9 | 39.0%  (23.5%,  56.0%) |
| P38 | 1 | 40.5%  (24.8%,  57.5%) | 2 | 73.4%  (56.9%,  86.8%) | 0 | 19.2%  (7.88%,  34.8%) | 25.9 | 28.2%  (14.5%,  44.9%) |
| P39 | 0 | 25.5%  (12.4%,  42.0%) | 0 | 20.3%  (8.60%,  36.1%) | 4 | 72.4%  (55.7%,  86.0%) | 33.2 | 70.7%  (53.9%,  84.7%) |
| P40 | 5 | 92.0%  (80.5%,  98.3%) | 3 | 90.9%  (78.6%,  97.8%) | 7 | 95.0%  (85.7%,  99.3%) | 29.1 | 46.3%  (30.1%,  63.1%) |
| P41 | 0 | 25.5%  (12.4%,  42.0%) | 0 | 20.3%  (8.60%,  36.1%) | 1 | 30.6%  (16.4%,  47.4%) | 35.4 | 81.0%  (65.4%,  92.2%) |
| P42 | 0 | 25.5%  (12.4%,  42.0%) | 0 | 20.3%  (8.60%,  36.1%) | 0 | 19.2%  (7.88%,  34.8%) | 27.9 | 39.1%  (23.6%,  56.1%) |
| P43 | 2 | 57.2%  (40.2%,  73.1%) | 1 | 45.8%  (29.6%,  62.6%) | 7 | 95.0%  (85.7%,  99.3%) | 47.7 | 99.5%*  (97.1%,  100%) |
| P44 | 0 | 25.5%  (12.4%,  42.0%) | 0 | 20.3%  (8.60%,  36.1%) | 0 | 19.2%  (7.88%,  34.8%) | 24.5 | 21.4%  (9.36%,  37.3%) |
| P45 | 1 | 40.5%  (24.8%,  57.5%) | 3 | 90.9%  (78.6%,  97.8%) | 2 | 44.4%  (28.3%,  61.3%) | 35.2 | 79.9%  (64.2%,  91.6%) |
| P46 | 5 | 92.0%  (80.5%,  98.3%) | 3 | 90.9%  (78.6%,  97.8%) | 7 | 95.0%  (85.7%,  99.3%) | 35.2 | 80.1%  (64.4%,  91.7%) |
| P47 | 1 | 40.5%  (24.8%,  57.5%) | 4 | 97.7%*  (91.5%,  99.8%) | 4 | 72.4%  (55.7%,  86.0%) | 30.8 | 56.9%  (40.0%,  72.9%) |
| P48 | 1 | 40.5%  (24.8%,  57.5%) | 0 | 20.3%  (8.60%,  36.1%) | 1 | 30.6%  (16.4%,  47.4%) | 26.0 | 28.5%  (14.7%,  45.2%) |

* <0.05, ** < 0.01

**Appendix C**

Composite cognitive scores, as illustrated in Tables 3, 4, 5, and 6, along with their corresponding z-scores.

|  | **Composite cognitive score** | **Corresponding z-score** |
| --- | --- | --- |
| **Table 3, 5 (Pre-TYB)** | 28.2 | -0.13 |
| **Table 3, 6 (Pre-TYB)** | 37.9 | 1.48 |
| **Table 4, 5 (Post-TYB)** | 31.5 | 0.27 |
| **Table 4, 6 (Post-TYB)** | 28.1 | -0.23 |
